# Editorial: Animal Transport and Related Management

**DOI:** 10.3389/fvets.2020.614317

**Published:** 2020-11-27

**Authors:** Mette S. Herskin, Todd Duffield

**Affiliations:** ^1^Department of Animial Science, Aarhus University, AU-FOULUM, Tjele, Denmark; ^2^University of Guelph, Ontario Western College, Population Medicine, Guelph, ON, Canada

**Keywords:** cull animal, space allowance, legislation, animal welfare, pigs, cattle

In modern livestock production, almost all animals are transported and often more than once. In addition, livestock transport journeys are increasing in distance due to structural development in the slaughter industry, leading to larger but fewer slaughterhouses. EU regulations, aiming to protect animals, specify minimum requirements for, for example, journey duration or space allowance. In other regions, such as North America, legislation also exists, but there are widespread differences between regions in terms of for example journey duration. In some countries, e.g., Canada, new national transport regulations have recently been written (2019), but these will not be fully enforced for a few years. However, scientific focus on animal transport is relatively new, which means that the majority of existing regulations and guidelines cannot be fully evidence based.

In their paper, Arndt et al. use image analysis to determine floor area covered (static space) by market weight pigs in different body positions, thereby targeting one area of the European Regulation (1) specifying space allowance for pigs during transportation. As reviewed by EFSA (2), loading density in trucks transporting animals influence animal welfare. Accordingly, European regulation (1) specifies that stocking density for pigs should not exceed 235 kg/m^2^ in order to ensure that all pigs are able to stand and lie in a natural body position. Based on analysis of 232 finishing pigs, the results show that the floor surface covered by the body of a pig depends on the body weight and on body posture. When lying in a full lateral position, a pig takes up 0.486 ± 0.040 m^2^. Using these results, Arndt et al. discuss whether compliance with Regulation (1) is possible by use of the current minimum space allowance, when modern hybrid pigs are transported to slaughter under commercial conditions, and call for studies of loading density in pigs to be able to include needs for dynamic and social space as well as to update the current legislation.

In Canada, Rioja-Lang et al. reviewed available scientific literature related to the welfare of pigs during transportation as part of the update of the codes of practice. The review focuses on priority welfare issues decided by National Farm Animal Care Council Scientific Committee, such as transport duration, time off feed and water, rest intervals, environmental conditions, and loading density, as single factors or in combination. The review is centered on market weight pigs (in Canada: 100–135 kg), thereby reflecting the current literature available. Rioja-Lang et al. adds a further component to the work by Arndt et al. described above and conclude that ambient conditions affect the impact of loading density. In general, however, greater loading density increases the risk of pigs becoming non-ambulatory or dying. The complexity is further increased by the suggestion that no simple relationship exists between journey duration and reduced welfare, as welfare may be reduced from both long and short journeys, resulting in fatigue/dehydration and acute stress, respectively. Based on available literature, Rioja-Lang et al. therefore cannot draw a clear conclusion on a maximum transport duration. Another major outcome of the paper by Rioja-Lang et al. is their call for studies focusing on other animal categories, such as weaned piglets or cull sows and boars, as these have only attracted limited scientific attention, thereby limiting the scope of identified priority welfare issues during transportation.

The remaining three papers of the Research Topic focus on cull animals. Two papers focus on dairy cows (Edwards-Callaway et al. and Dahl-Pedersen et al.) and one on sows. Despite the different animal species, management of cull cows and sows share many similarities (3, 4). In their perspective paper, Edwards-Callaway et al. describe the process of sending dairy cows to slaughter in the US, including discussion of animal welfare and fitness for transport. The authors conclude that although decisions begin on the dairy farm, all stakeholders in the livestock market, transport, and slaughter process have responsibility and ability to protect welfare of dairy cattle. As part of the discussion, attention is drawn to differences between US and European conditions.

The latter is described by Dahl-Pedersen et al., examining risk factors for deterioration of the clinical condition of cull dairy cows during transport to slaughter in Denmark. Here, national legislation stipulates that cull dairy cows cannot be transported for more than 8 h. However, even during this interval, where cows were transported directly from farm of origin to slaughterhouse, Dahl-Pedersen et al. find significant deterioration in the clinical condition of the cows, as indicated by an increased proportion of cows with increased gait score and wounds. Among identified risk factors for the increase in gait score were low body condition, digital dermatitis, and pelvic asymmetry. No risk factors for increased occurrence of wounds were identified. Based on wording from the European Regulation (1), stating “all animals shall be transported in conditions guaranteed not to cause them injury,” Dahl-Pedersen et al. discuss whether the increased proportion of cows with wounds upon arrival at the slaughterhouse could be interpreted as violation of the regulation. This would, however, depend upon the chosen definition of “injury,” which is not specified in the regulation.

In a study using similar methodology, but focusing on cull sows, Thodberg et al. come to similar conclusions - that the clinical condition of sows deteriorates during transportation (study limited to 0–8 h). However, in contrast to the dairy cow findings, risk factors for deterioration of the condition of sows were not related to animal characteristics but were associated with the journey, such as temperature and duration - often interacting - and duration of stationary periods. These findings add further complexity to the concept of fitness for transport, a concept considered crucial for animal welfare during transportation (5), but with no scientific definition and where only little research has been done.

Taken together, papers constituting this Research Topic have brought new knowledge to the area of animal transportation. However, as exemplified with the concept of fitness for transport, another main outcome of the Research Topic is awareness of the lack of knowledge within this area of high importance for welfare of farm animals.

## Author Contributions

MH and TD contributed to the content and style of the editorial.

## Conflict of Interest

The authors declare that the research was conducted in the absence of any commercial or financial relationships that could be construed as a potential conflict of interest.

## References

[1] EC ANON. Council Regulation (EC) NO 1/2005 of 22 December 2004 on the Protection of Animals During Transport and Related Operations and Amending Directives 64/432/EEC and 93/119/EC and Regulation (EC) NO 1255/97. (2005). Available online at: https://eurlex.europa.eu/legal-content/EN/TXT/PDF/?uri=CELEX:32005R0001&from=da (accessed October 2020).

[2] EFSA Scientific opinion concerning the welfare of animals during transport. EFSA J. (2011) 9:166 10.2903/j.efsa.2011.1966

[3] Stojkov J Bowers G Draper M Duffield T Duivenvoorden P Groleau M. Management of cull cows – consensus of an expert consultation in Canada. J Dairy Sci. (2018) 101:11170–4. 10.3168/jds.2018-14919 30243623

[4] Herskin MS Aaslyng MD Anneberg I Thomsen PT Gould LM Thodberg K. Significant variation in the management of cull cows before transport for slaughter: results from a survey of Danish pig farmers. Vet Rec. (2020). 186:185–92. 10.1136/vetrec-2019-105671 31941806

[5] Cockram MS. Fitness of animals for transport to slaughter. Can Vet J. (2019) 60:423–9. 30992599 PMC6417610

